# Fish species richness in the Terminos Lagoon: An occurrence data compilation of four sampling campaigns along a multidecadal series

**DOI:** 10.3897/BDJ.9.e65317

**Published:** 2021-05-21

**Authors:** Carlos E. Paz-Ríos, Atahualpa Sosa-López, Yassir E. Torres-Rojas, Julia Ramos-Miranda, Rodolfo E. del Río-Rodríguez

**Affiliations:** 1 Instituto de Ecologia, Pesquerias y Oceanografia del Golfo de Mexico, Universidad Autonoma de Campeche (EPOMEX-UAC), Campus VI, 24029 San Francisco de Campeche, Campeche, Mexico Instituto de Ecologia, Pesquerias y Oceanografia del Golfo de Mexico, Universidad Autonoma de Campeche (EPOMEX-UAC) Campus VI, 24029 San Francisco de Campeche, Campeche Mexico

**Keywords:** biodiversity, Campeche, coastal lagoon, estuary, fish occurrence, fishery resource, Gulf of Mexico

## Abstract

**Background:**

Here we present an occurrence dataset that contributes to the knowledge of tropical fish distribution in coastal habitats from the Terminos Lagoon Flora and Fauna Protection Area, one of the largest lagoon ecosystems in the Gulf of Mexico. Fish are high biomass keystone species in the Terminos Lagoon which provide socio-economic and ecosystem services. An initiative in 1980 was carried out to systematically sample the fish community of Terminos Lagoon for an annual cycle; the effectiveness of its results led to replicate in the lagoon the same sampling design for three more campaigns in 1998, 2010 and 2016. Constituted as a Flora and Fauna Protection Area in 1994, the Terminos Lagoon has received many efforts to inventory its biodiversity, particularly on the fish community since the 70s; however, these studies did not have consistent survey protocols, nor the longevity of the present study, which was over four decades.

**New information:**

A total of 141 fish species, belonging to 90 genera, 49 families, 20 orders and two classes are presented in this study. Information on fish species occurrence data is provided corresponding to the Terminos Lagoon coastal ecosystem, southern Gulf of Mexico, assembled from four time periods at 1980, 1998, 2010 and 2016. The records form part of a consistently homogeneous database compilation, mostly derived from the research programme's sampled material on tropical fishery resources of the “Instituto de Ecologia, Pesquerias y Oceanografia del Golfo de Mexico (EPOMEX)”. The current dataset represents the first and most comprehensive online open-access source of information concerning the fish community occurring along and wide of the Terminos Lagoon ecosystem, with 1,249 data records and a total count of 48,717 organisms. Data are available through the Ocean Biodiversity Information System (OBIS).

## Introduction

Terminos Lagoon is a tropical coastal aquatic system with high biodiversity and abundant natural resources (Rivera-Arriaga and Villalobos-Zapata 2005). This wealthy coastal area represents for the population an important source of productive activities from goods directly realised (e.g. fishing, ecotourism) and of environmental health from ecosystem services indirectly earned (e.g. material recycling, carbon sink). In this respect, the fish community could provide a valuable balance in food webs within and between ecosystems via nutrient flow, due to differential species migration patterns (Abascal-Monroy et al. 2016). In addition, because numerous fish species are commercially important for local populations settled around the lagoon, the fish’s spatiotemporal dynamics could impact on the economic welfare by differential exploitation levels in the lagoon habitats (Campos-Flores and Crespo-Guerrero 2018). Fish community along with environmental conditions have undergone changes in the Terminos Lagoon, observed as temporal turnovers in the taxonomic composition and functional traits that suggest a restructuring in the lagoon ecological status (Ramos-Miranda et al. 2015).

The fish community from the Terminos Lagoon has been sampled extensively in space and time by virtue of the academic initiatives of the research programme on tropical fishery resources from the “Instituto de Ecologia, Pesquerias y Oceanografia del Golfo de Mexico (EPOMEX)”, forming a wide range of biological and environmental data, useful for monitoring the lagoon ecological status. Within the framework of this sampling effort, information on the fish community has been obtained systematically and consistently. Data was collected during the years 1980, 1998, 2010 and, more recently, in 2016. The primer for this long-term study programme highlights the research of Dr. Alejandro Yáñez Arancibia, who was one of the most renowned academics in Mexico on coastal ecology and conservation, contributing and compiling the first compendium on natural resources and ecological processes of the Terminos Lagoon (Yáñez-Arancibia and Day 1988). The present study represents a fish occurrence data compilation that makes significative progress since that first ichthyological survey by recording new temporal ranges of distribution and making it available by using biodiversity data services. In the context of data papers, an occurrence record refers to the timely georeferenced presence of a species in a location, with a link to openly accessible metadata (Penev et al. 2017).

The extensive work of multiple institutions helped to provide further a wide variety of information on biological, ecological and socioeconomic aspects of the ichthyofauna in the Terminos Lagoon (e.g. Sánchez 1994, Castro-Aguirre et al. 1999, Guevara et al. 2007, Amador-del Angel et al. 2012, Ruíz-Marín et al. 2012, García-Ríos et al. 2013, Campos-Flores and Crespo-Guerrero 2018). Simultaneously, the academic production on fish topics was highlighted from the joint collaborations between the EPOMEX Institute with national and international institutions, including, but not limited to, the following universities: “Universidad Autonoma Metropolitana-Xochimilco” (e.g. Ayala-Pérez et al. 2001, Ayala-Pérez et al. 2003, Ayala-Pérez et al. 2008) and “Universite Montpellier II” (e.g. Ramos-Miranda et al. 2005a, Ramos-Miranda et al. 2005b, Sosa-López et al. 2005, Sosa-López et al. 2007, Villéger et al. 2010), describing spatio-temporal trends in the species composition, associated with the regional weather seasons and environmental variability of habitats. With the bulk of new data generated by the EPOMEX Institute, an updated compendium on the fish community and environmental conditions in the Terminos Lagoon was published (Ramos-Miranda et al. 2006), including the spatial mapping of ecologically important species in the lagoon. After a few more years, a national initiative for documenting the biodiversity of Mexico led to another effort by the Institute, contributing with a synthesis on marine and estuarine fish present, providing an inventory of species for the Terminos Lagoon (Ramos-Miranda et al. 2010). After this work, contributions on diverse aspects of the ichthyofauna in the Terminos Lagoon were continually provided (e.g. Ramos-Miranda et al. 2015, Sirot et al. 2015a, Sirot et al. 2015b, Dorantes-Hernández et al. 2020, Escobar-Toledo et al. 2017), highlighting two of the most recent books on the Lagoon, a valuable illustrated catalogue covering the fish composition (Ayala-Pérez et al. 2015) and a new compendium on current socio-environmental aspects provided (Ramos-Miranda and Villalobos-Zapata 2015).

Recognised in 1994 as a Flora and Fauna Protection Area, the Terminos Lagoon (along with the Campeche continental shelf) maintains numerous commercially important fisheries which employ thousands of people in the southern Gulf of Mexico. However, the historical trend in the fish abundance and biomass in Terminos Lagoon might suggest an overall decrease in catch volumes (Ramos-Miranda et al. 2015). Therefore, understanding the long-term dynamics of this resource is essential in determining the present community assemblage and current magnitude species richness in order to support and maintain livelihoods in the region.

## General description

### Purpose

The objective of the present study is to document the fish species richness by sharing information on a species occurrence dataset in the Terminos Lagoon, one of the largest and most productive lagoon-estuarine ecosystems in the Gulf of Mexico. To achieve this objective, species occurrence data from four methodologically consistent sampling campaigns were assembled, covering four decades of ecological surveys along and wide of the Lagoon.

## Project description

### Title

Terminos Lagoon Fish Occurrence.

### Personnel

Atahualpa Sosa López, Julia Ramos Miranda, Yassir Edén Torres Rojas, Domingo Flores Hernández, Francisco Gómez Criollo, Luis Alejandro Yáñez Arancibia, Ana Laura Lara Domínguez, Luis Amado Ayala Pérez, Hernán Álvaréz Guillén, Sebastien Villèger, David Mouillot, Rodolfo Enrique del Río Rodríguez, Carlos Enrique Paz Ríos.

### Design description

Terminos Lagoon shows a high spatiotemporal variability in its biophysical characteristics; hence, four zones have been determined by ecological studies to characterise different environmental conditions and fish assemblages. The project design is aimed to provide species occurrence data by zone, so four coordinates were represented as different centroids, determined from three to five sampling sites per zone for representing each one of these. In the different sampling campaigns, the same methodological protocols for collecting fish samples was carried out through the four time periods.

### Funding

The resources to undertake research in the Lagoon have been received from diverse academic projects: “Mecanismos de produccion en Ecosistemas Lagunares Costeros, Laguna de Terminos, Mexico y Laguna de Arcachon, Francia, UNAM-CONACYT-Universidad de Burdeaux QCMACFR-001698”; Evaluacion del camaron blanco (*Penaeus setiferus*) y de las comunidades nectonicas de la Laguna de Terminos-Sonda de Campeche, Mexico: interaccion de los impactos ambientales y pesqueros, SISIERRA ALIM/97”; “Ecologia del Paisaje y Diagnostico Ambiental del ANP de la Laguna de Terminos, P/SISIERRA 2000706030”; “Long-term effects of environmental changes on the nekton biodiversity and the functioning of tropical estuaries, BIODIVNEK ANR-CONACYT C0004-2009-01-000000000111465”; and “Analisis de δ13C y δ18O en otolitos de peces marinos presentes en el Area de Proteccion de Flora y Fauna Laguna de Terminos, Campeche: indicadores del cambio climatico, FONSEC SEMARNAT-CONACYT 000000000263401”.

## Sampling methods

### Study extent

The dataset contains information on fish collected (juvenile and adult stages) in four different campaigns: the first one in February 1980 – January 1981, the second one in February 1998 – January 1999, the third one in November 2010 – October 2011 and the fourth one in September 2016 – August 2017. In each sampling campaign, the lagoon was visited in a N-S direction, as well as an E-W direction in order to increase the chances of capturing the greatest species diversity, taking samples from different environmental conditions, such as habitats dominated by seagrasses or sand beds and near to river mouths or inlets connected to the sea with different salinity concentrations. Each sampling campaign encompassed an annual period that covered the three regional weather seasons: dry, rainy, winter storms.

### Sampling description

Fish samples were collected with a shrimp trawl net. This fishing gear was an active method consisting of a 5-m long net with a mouth opening diameter of 2.5 m and mesh size of 19 mm. One net per site was trawled at a speed of ~ 4.6 km h^-1^ (2.5 knots) for 12 min, covering an area of 2000 m^2^ each trawl. Fish samples were placed in plastic bags and stored in ice during the sampling campaigns. In the laboratory, fish were sorted and identified to the species category. Organisms from the last three sampling campaign were transferred to 70% ethanol and provisionally stored at the fisheries laboratory of the EPOMEX Institute.

### Quality control

Fish species were sorted and identified to the species taxonomical category, using specialised literature, such as guides and keys for identification, for example, Fischer (1978), Hildebrand (1943), Castro-Aguirre (1978), Reséndez (1981). The taxonomical arrangement used herein follows Fricke et al. (2021). Species names were matched using the *Taxon Match* tool in the World Register of Marine Species (WoRMS) in order to obtain an unique identifier, such as the *AphiaID.* Centroid points in the Lagoon, available in the Data resources (see below) were georeferenced and displayed in different maps using different software (i.e. ArcView GIS and Google Maps).

### Step description

**Exploratory analysis of the fish occurrence data**

**Species richness**: The total number of species, with regards to time, was evaluated by the sampling effort through the four sampling campaigns, using a species accumulation curve with interpolation-extrapolation of the Hill number for incidence data (Chao and Jost 2012). This species accumulation curve estimates the potential species richness and the sample coverage, including, in the present analysis, an additional sampling effort for simulating an extra campaign. Analyses were undertaken using the package iNEXT in R (Hsieh et al. 2016).

**Temporal pattern**: Temporal changes in composition and abundance (root square transformation) through the time of commercially important species, according to Ayala-Pérez et al. (2015), were explored using a heatmap plot, classified by the different sampling year periods and zone in the dataset. The heatmap plot was undertaken using the statistical software PRIMER (Clarke and Gorley 2006).

## Geographic coverage

### Description

**Figure 1.**
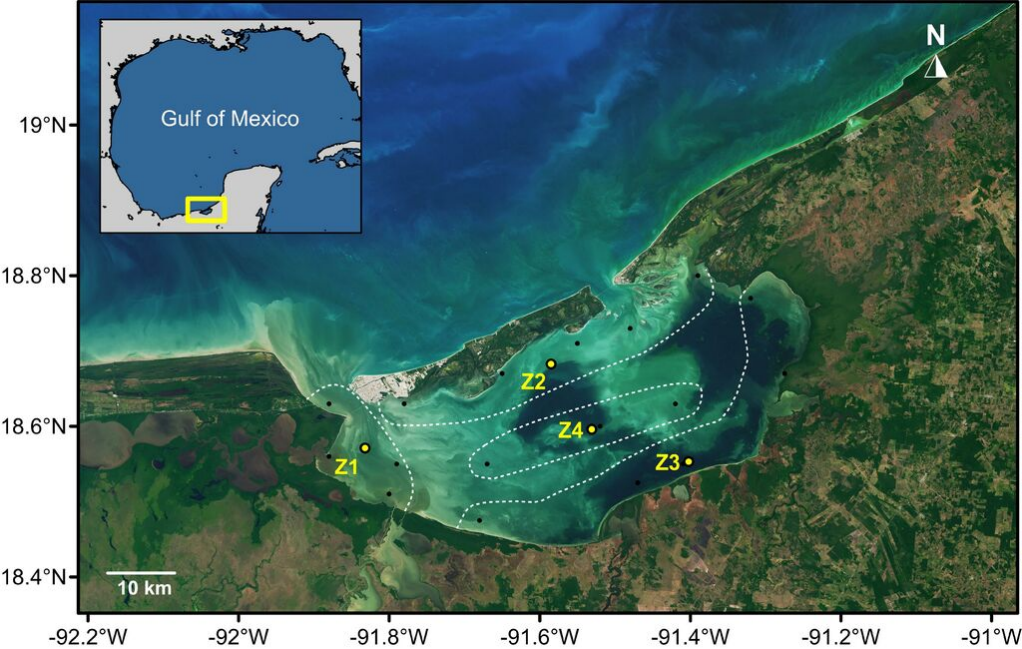
Location of sampling sites and the four centroids per zone in the Terminos Lagoon, Campeche, Mexico. Zones based on classification by Villéger et al. (2010). Image source: NASA Earth Observatory, acquired by Joshua Stevens on 20 January 2017, by the Operation Land Imager on Landsat 8 from the U.S. Geological Survey.

The Terminos Lagoon is situated in the Mexican littoral zone of Campeche (Fig. 1), forming part of the Tropical North-western Atlantic’s marine ecoregion 69 (Southern Gulf of Mexico) (Spalding et al. 2007). Terminos Lagoon is a shallow water body (3.5 m depth) that covers an approximate water surface area of ~ 1700 km^2^; it is a lagoon-estuarine system, separated from the Gulf of Mexico by the Carmen Island, connected to the sea by two permanently open inlets: Puerto Real inlet on its eastern side and Carmen inlet on its western side, receiving freshwater from three streams on its southern part: Palizada River, Chumpan River and Candelaria River (Ramos-Miranda et al. 2005a).

### Coordinates

18°40' N and 18°90' N Latitude; 92°00' W and 91°20' W Longitude.

## Taxonomic coverage

### Description

The dataset contains occurrence records from organisms belonging to the class Actinopteri and Elasmobranchii (Paz-Ríos and Sosa-López 2021). The complete taxonomic inventory is composed of 141 species, 90 genera, 49 families and 20 orders (Table 1). The class Actinopteri is composed of 134 species, 84 genus, 44 families and 18 orders, whereas the class Elasmobranchii is composed of seven species, six genus, five families and two orders. Regardless of class, 11 families presented the highest numbers of species, accounting for the 57% of all the fauna in the lagoon: Carangidae (12 spp.), Clupeidae, (7 spp.), Engraulidae (5 spp.), Gerreidae (6 spp.), Haemulidae (6 spp), Lutjanidae (5 spp.), Sciaenidae (13 spp.), Sparidae (5 spp.), Syngnathidae (6 spp.), Tetraodontidae (9 spp.) and Triglidae (7 spp).

**Figure 2.**
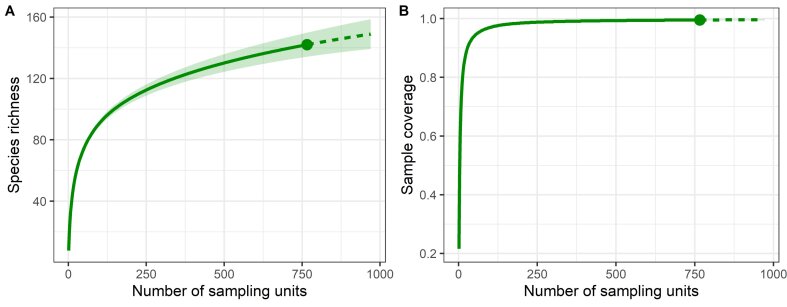
Species accumulation curve (A) and sample completeness (B), based on the interpolation (solid line) and extrapolation (dashed line) analyses of fish species richness in the Terminos Lagoon, Campeche, Mexico. The knot on curves indicates the observed species richness.

**Species richness**: The species accumulation curve showed a weak slope at the observed species richness (141 spp.) as a function of the number of sampling units through the four periods of study (Fig. 2A). The data extrapolation for the fish species richness showed a slight tendency to increase in the number of species undetected, suggesting an estimated species richness of 148 ssp. (159–138 spp. 95% CL) after including a number of sampling units that simulated an extra campaign. The sample coverage of the four sampling periods analysed was higher than 0.9 (Fig. 2B), suggesting that unsampled species would represent a coverage deficit lower than 10% to reach an asymptote in the sampling effort. The representative nature of the analysed species richness roughly agrees with the numbers previously reported for the lagoon, ranking the extreme values for fish species observed at 117–149 nominal species (Yáñez-Arancibia et al. 1988, Ramos-Miranda et al. 2015).

**Figure 3.**
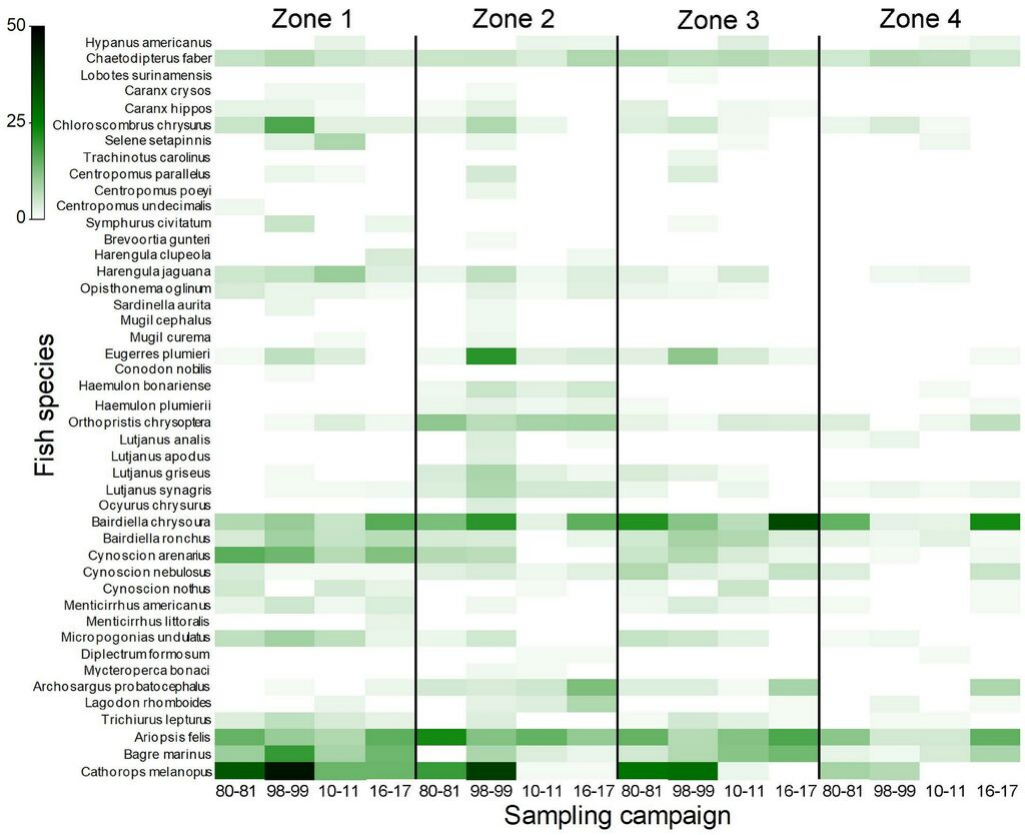
Composition and abundance (root square transformation) of commercially important fish species after Ayala-Pérez et al. (2015) recorded by the sampling campaign and zone in the Terminos Lagoon, Campeche, Mexico.

**Temporal pattern**: As part of the fish community recorded for the Terminos Lagoon, 45 spp. are considered commercially important (Fig. 3). Occurrence and abundance data on this resource showed that most of the species display a highly dynamic distribution through the sampling years and zones, but in general, zone 4 presented lower species abundance values. Several fish species were poorly represented and recorded at one or two sampling campaigns with low abundance, such as *Brevoortia gunteri*, *Sardinella aurita*, *Centropomus poeyi*, *Centropomus undecimalis* or *Ocyurus chrysurus*. Other locally exploited species were maintained relatively consistently through the sampling years, such as some Clupeidae (e.g. *Herengula jaguana*), Ephippida (*Chaetodipterus faber*), Sciaenidae (e.g. *Bairdiella chrysoura*, *Bairdiella ronchus*, *Cynoscion arenarius*, *Cynoscion nebulosus*) or Ariidae (e.g. *Ariopsis felis*, *Bagre marinus*, *Cathorops melanopus*).

## Temporal coverage

### Notes

1 February 1980 – 17 August 2017.

## Usage licence

### Usage licence

Other

### IP rights notes

Creative Commons Attribution Non Commercial (CC-BY-NC) 4.0 License.

## Data resources

### Data package title

Fish occurrence data in the Terminos Lagoon, Campeche.

### Resource link

http://ipt.iobis.org/caribbeanobis/archive.do?r=fish_campeche

### Alternative identifiers

http://ipt.iobis.org/caribbeanobis/resource?r=fish_campeche

### Number of data sets

1

### Data set 1.

#### Data set name

Fish occurrence data in the Terminos Lagoon, Campeche.

#### Data format

Darwin Core Archive (DwC-A).

#### Number of columns

28

#### Character set

UTF-8.

#### Download URL

https://www.gbif.org/dataset/1f7bb798-150d-44c2-b7a6-372f341e5ff4

#### Data format version

1.3

#### Description

The dataset presents an occurrence data sheet with 28 columns including information for 1,249 records (Paz-Ríos and Sosa-López 2021).

**Data set 1.** 

Column label	Column description
eventID	An identifier for the set of information associated with an Event (something that occurs at a place and time).
parentEventID	An event identifier for the super-event which is composed of one or more sub-sampling events.
samplingProtocol	The name of, reference to, or description of the method or protocol used during an Event.
eventDate	The date-time or interval during which an Event occurred.
identifiedBy	A list (concatenated and separated) of names of people, groups or organisations who assigned the Taxon to the subject.
waterbody	The name of the water body in which the Location occurs.
country	The name of the country or major administrative unit in which the Location occurs.
countryCode	The standard code for the country in which the Location occurs.
decimalLatitude	The geographic latitude (in decimal degrees, using the spatial reference system given in geodeticDatum) of the geographic centre of a Location.
decimalLongitude	The geographic longitude (in decimal degrees, using the spatial reference system given in geodeticDatum) of the geographic centre of a Location.
geodeticDatum	Spatial reference system (SRS) upon which the geographic coordinates given in decimalLatitude and decimalLongitude are based.
occurrenceID	An identifier for the Occurrence (as opposed to a particular digital record of the occurrence). In the absence of a persistent global unique identifier, construct one from a combination of identifiers in the record that will most closely make the occurrenceID globally unique.
individualCount	The number of individuals represented present at the time of the Occurrence.
occurrenceStatus	A statement about the presence or absence of a Taxon at a Location.
preparations	A list (concatenated and separated) of preparations and preservation methods for a specimen.
associatedReferences	A list (concatenated and separated) of identifiers (publication, bibliographic reference, global unique identifier, URI) of literature associated with the Occurrence.
institutionCode	The name (or acronym) in use by the institution having custody of the object(s) or information referred to in the record.
datasetName	The name identifying the dataset from which the record was derived.
basisOfRecord	The specific nature of the data record.
dataGeneralisations	Actions taken to make the shared data less specific or complete than in its original form. Suggests that alternative data of higher quality may be available on request.
scientificNameID	An identifier for the nomenclatural (not taxonomic) details of a scientific name.
scientificName	The full scientific name (with authorship and date information if known. => add to scientificNameAuthorship).
kingdom	The full scientific name of the kingdom in which the taxon is classified.
phylum	The full scientific name of the phylum or division in which the taxon is classified.
class	The full scientific name of the class in which the taxon is classified.
order	The full scientific name of the order in which the taxon is classified.
family	The full scientific name of the family in which the taxon is classified.
taxonRank	The taxonomic rank of the most specific name in the scientificName.

## Additional information

**Ethics statement**: The authors declare that they have no conflict of interest. Applicable international and national guidelines for the care and use of animals were followed by the authors. All necessary permits for sampling and observational field studies have been obtained by the authors from the competent authorities.

## Figures and Tables

**Table 1.** Taxonomic classification of the fish community from the Terminos Lagoon, Campeche, Mexico. * Commercially important species after Ayala-Pérez et al. (2015). ~ Reported species in freshwater habitats after Fricke et al. (2021). Sampling campaigns: 1, 1980-1981; 2, 1998-1999; 3, 2010-2011; 4, 2016-2017.

Class	Order	Family	Species	1	2	3	4
Elasmobranchii	Myliobatiformes	Dasyatidae	*Dasyatis hastata* (DeKay, 1842)	●			
			*Hypanus americanus (Hildebrand & Schroeder, 1928)			●	●
			~*Hypanus sabinus* (Lesueur, 1824)	●	●	●	●
		Gymnuridae	*Gymnura micrura* (Bloch & Schneider, 1801)			●	●
		Potamotrygonidae	*Styracura schmardae* (Werner, 1904)		●		●
		Urotrygonidae	*Urobatis jamaicensis* (Cuvier, 1816)	●	●	●	●
	Rhinopristiformes	Rhinobatidae	*Pseudobatos lentiginosus* (Garman, 1880)			●	●
Actinopteri	Acanthuriformes	Ephippidae	*~*Chaetodipterus faber* (Broussonet, 1782)	●	●	●	●
		Lobotidae	*~*Lobotes surinamensis* (Bloch, 1790)		●		
	Anguilliformes	Muraenidae	*Gymnothorax nigromarginatus* (Girard, 1858)			●	
			*Gymnothorax saxicola* Jordan & Davis, 1891		●		
		Ophichthidae	*Ophichthus gomesii* (Castelnau, 1855)	●	●		
	Aulopiformes	Synodontidae	*Synodus foetens* (Linnaeus, 1766)	●	●	●	●
	Batrachoidiformes	Batrachoididae	~*Opsanus beta* (Goode & Bean, 1880)	●	●	●	●
			*Porichthys porosissimus* (Cuvier, 1829)	●	●	●	
	Beloniformes	Belonidae	~*Strongylura notata* (Poey, 1860)		●		
		Hemiramphidae	~*Chriodorus atherinoides* Goode & Bean, 1882	●			●
	Blenniiformes	Blenniidae	*Hypleurochilus geminatus* (Wood, 1825)				●
		Labrisomidae	*Paraclinus fasciatus* (Steindachner, 1876)				●
	Carangiformes	Achiridae	~*Achirus lineatus* (Linnaeus, 1758)	●	●	●	●
			~*Trinectes maculatus* (Bloch & Schneider, 1801)	●	●	●	●
		Bothidae	*Bothus ocellatus* (Agassiz, 1831)	●			
		Carangidae	*Caranx crysos (Mitchill, 1815)		●	●	
			*~*Caranx hippos* (Linnaeus, 1766)	●	●	●	●
			~*Caranx latus* Agassiz, 1831			●	
			*Caranx ruber* (Bloch, 1793)			●	
			*Chloroscombrus chrysurus (Linnaeus, 1766)	●	●	●	●
			*Hemicaranx amblyrhynchus* (Cuvier, 1833)		●	●	●
			~*Oligoplites saurus* (Bloch & Schneider, 1801)	●	●	●	
			*Selene setapinnis (Mitchill, 1815)		●	●	
			*Selene vomer* (Linnaeus, 1758)	●	●	●	●
			*Trachinotus carolinus (Linnaeus, 1766)		●		
			*Trachinotus falcatus* (Linnaeus, 1758)		●		
			*Trachinotus goodei* Jordan & Evermann, 1896		●		
		Centropomidae	*~*Centropomus parallelus* Poey, 1860		●	●	
			*~*Centropomus poeyi* Chávez, 1961		●		
			*~*Centropomus undecimalis* (Bloch, 1792)	●			
		Cynoglossidae	*Symphurus civitatum Ginsburg, 1951		●		●
			~*Symphurus plagiusa* (Linnaeus, 1766)	●	●	●	●
		Paralichthyidae	*Ancylopsetta quadrocellata* Gill, 1864	●		●	
			~*Citharichthys spilopterus* Günther, 1862	●	●	●	●
			*Etropus crossotus* Jordan & Gilbert, 1882	●	●	●	●
			*Syacium gunteri* Ginsburg, 1933			●	
		Polynemidae	*Polydactylus octonemus* (Girard, 1858)	●	●	●	●
		Sphyraenidae	*Sphyraena barracuda* (Edwards, 1771)			●	
	Cichliformes	Cichlidae	~*Mayaheros urophthalmus* (Günther, 1862)	●	●	●	●
	Clupeiformes	Clupeidae	*~*Brevoortia gunteri* Hildebrand, 1948		●		
			~*Dorosoma anale* Meek, 1904			●	
			~*Dorosoma petenense* (Günther, 1867)		●	●	
			*Harengula clupeola (Cuvier, 1829)				●
			*~*Harengula jaguana* Poey, 1865	●	●	●	●
			*Opisthonema oglinum (Lesueur, 1818)	●	●	●	●
			*Sardinella aurita Valenciennes, 1847		●		
		Engraulidae	~*Anchoa hepsetus* (Linnaeus, 1758)	●	●	●	●
			*Anchoa lamprotaenia* Hildebrand, 1943	●			●
			*Anchoa lyolepis* (Evermann & Marsh, 1900)			●	
			~*Anchoa mitchilli* (Valenciennes, 1848)	●	●	●	●
			*Cetengraulis edentulus* (Cuvier, 1829)	●	●	●	●
	Cyprinodontiformes	Fundulidae	~*Lucania parva* (Baird & Girard, 1855)				●
	Elopiformes	Elopidae	*Elops saurus* Linnaeus, 1766		●		
	Gobiiformes	Eleotridae	~*Eleotris pisonis* (Gmelin, 1789)				●
		Gobiidae	~*Bathygobius soporator* (Valenciennes, 1837)		●		
			~*Gobionellus oceanicus* (Pallas, 1770)	●	●	●	●
			*Gobiosoma longipala* Ginsburg, 1933				●
	Mugiliformes	Mugilidae	*~*Mugil cephalus* Linnaeus, 1758		●		
			*~*Mugil curema* Valenciennes, 1836		●	●	
	Perciformes	Scaridae	*Nicholsina usta* (Valenciennes, 1840)	●	●		
		Scorpaenidae	*Scorpaena brasiliensis* Cuvier, 1829			●	
			*Scorpaena plumieri* Bloch, 1789	●		●	●
		Triglidae	*Prionotus beanii* Goode, 1896	●			
			*Prionotus carolinus* (Linnaeus, 1771)	●	●	●	
			*Prionotus martis* Ginsburg, 1950			●	
			*Prionotus punctatus* (Bloch, 1793)	●		●	
			*Prionotus rubio* Jordan, 1886			●	●
			*Prionotus scitulus* Jordan & Gilbert, 1882	●	●	●	●
			*Prionotus tribulus* Cuvier, 1829	●		●	●
		Gerreidae	~*Diapterus auratus* Ranzani, 1842		●	●	●
			~*Diapterus rhombeus* (Cuvier, 1829)	●	●	●	●
			~*Eucinostomus argenteus* Baird & Girard, 1855	●	●	●	●
			~*Eucinostomus gula* (Quoy & Gaimard, 1824)	●	●	●	●
			~*Eucinostomus melanopterus* (Bleeker, 1863)	●	●		●
			*~*Eugerres plumieri* (Cuvier, 1830)	●	●	●	●
		Haemulidae	*Anisotremus virginicus* (Linnaeus, 1758)				●
			*~*Conodon nobilis* (Linnaeus, 1758)		●		
			*Haemulon aurolineatum* Cuvier, 1830		●	●	●
			*Haemulon bonariense Cuvier, 1830	●	●	●	●
			*Haemulon plumierii (Lacepède, 1801)	●	●	●	●
			*~*Orthopristis chrysoptera* (Linnaeus, 1766)	●	●	●	●
		Lutjanidae	*~*Lutjanus analis* (Cuvier, 1828)	●	●		●
			*Lutjanus apodus (Walbaum, 1792)		●		
			*~*Lutjanus griseus* (Linnaeus, 1758)	●	●	●	●
			*Lutjanus synagris (Linnaeus, 1758)	●	●	●	●
			*Ocyurus chrysurus (Bloch, 1791)		●		
		Sciaenidae	*~*Bairdiella chrysoura* (Lacepède, 1802)	●	●	●	●
			*Bairdiella ronchus (Cuvier, 1830)	●	●	●	●
			*Corvula batabana* (Poey, 1860)				●
			*~*Cynoscion arenarius* Ginsburg, 1930	●	●	●	●
			*~*Cynoscion nebulosus* (Cuvier, 1830)	●	●	●	●
			*Cynoscion nothus (Holbrook, 1848)	●		●	●
			*Menticirrhus americanus (Linnaeus, 1758)	●	●	●	●
			*Menticirrhus littoralis (Holbrook, 1847)				●
			*Menticirrhus saxatilis* (Bloch & Schneider, 1801)	●	●	●	●
			~*Micropogonias furnieri* (Desmarest, 1823)			●	●
			*~*Micropogonias undulatus* (Linnaeus, 1766)	●	●	●	●
			*Odontoscion dentex* (Cuvier, 1830)	●			
			~*Stellifer lanceolatus* (Holbrook, 1855)	●	●	●	●
		Serranidae	*Diplectrum bivittatum* (Valenciennes, 1828)			●	
			*Diplectrum formosum (Linnaeus, 1766)			●	●
			*Epinephelus itajara* (Lichtenstein, 1822)		●		
			*Mycteroperca bonaci (Poey, 1860)		●	●	
		Sparidae	*~*Archosargus probatocephalus* (Walbaum, 1792)	●	●	●	●
			*Archosargus rhomboidalis* (Linnaeus, 1758)	●	●	●	●
			*Calamus penna* (Valenciennes, 1830)		●		
			*~*Lagodon rhomboides* (Linnaeus, 1766)		●	●	●
			*Stenotomus caprinus* Jordan & Gilbert, 1882				●
	Scombriformes	Stromateidae	*Peprilus paru* (Linnaeus, 1758)		●	●	
		Trichiuridae	*Trichiurus lepturus Linnaeus, 1758	●	●	●	●
	Siluriformes	Ariidae	*~*Ariopsis felis* (Linnaeus, 1766)	●	●	●	●
			*Bagre marinus (Mitchill, 1815)	●	●	●	●
			*~*Cathorops melanopus* (Günther, 1864)	●	●	●	●
	Syngnathiformes	Dactylopteridae	*Dactylopterus volitans* (Linnaeus, 1758)			●	
		Syngnathidae	*Hippocampus erectus* Perry, 1810	●	●	●	●
			*Hippocampus zosterae* Jordan & Gilbert, 1882				●
			*Syngnathus floridae* (Jordan & Gilbert, 1882)				●
			~*Syngnathus fuscus* Storer, 1839			●	
			*Syngnathus louisianae* Günther, 1870	●	●		●
			~*Syngnathus scovelli* (Evermann & Kendall, 1896)	●	●	●	●
	Tetraodontiformes	Diodontidae	*Chilomycterus schoepfii* (Walbaum, 1792)	●	●	●	●
		Monacanthidae	*Aluterus schoepfii* (Walbaum, 1792)	●		●	●
			*Monacanthus ciliatus* (Mitchill, 1818)		●	●	
			*Stephanolepis hispida* (Linnaeus, 1766)	●	●	●	●
		Ostraciidae	*Acanthostracion quadricornis* (Linnaeus, 1758)	●	●	●	●
		Tetraodontidae	*Lagocephalus laevigatus* (Linnaeus, 1766)	●	●	●	
			*Sphoeroides greeleyi* Gilbert, 1900	●	●	●	●
			*Sphoeroides maculatus* (Bloch & Schneider, 1801)			●	
			*Sphoeroides marmoratus* (Lowe, 1838)	●			
			*Sphoeroides nephelus* (Goode & Bean, 1882)	●	●	●	●
			*Sphoeroides pachygaster* (Müller & Troschel, 1848)			●	
			*Sphoeroides parvus* Shipp & Yerger, 1969			●	●
			*Sphoeroides spengleri* (Bloch, 1785)	●	●	●	●
			~*Sphoeroides testudineus* (Linnaeus, 1758)	●	●	●	●
